# Hospital‐treated infections and the risk and prognosis of amyotrophic lateral sclerosis: A population‐based study

**DOI:** 10.1111/joim.70008

**Published:** 2025-08-05

**Authors:** Yihan Hu, Charilaos Chourpiliadis, Caroline Ingre, Viktor H. Ahlqvist, Jiangwei Sun, Huan Song, Yudi Pawitan, Fredrik Piehl, Fang Fang

**Affiliations:** ^1^ Institute of Environmental Medicine Karolinska Institutet Stockholm Sweden; ^2^ Department of Clinical Neuroscience Karolinska Institutet Stockholm Sweden; ^3^ Department of Neurology Karolinska University Hospital Stockholm Sweden; ^4^ Department of Biomedicine Aarhus University Aarhus Denmark; ^5^ Department of Medical Epidemiology and Biostatistics Karolinska Institutet Stockholm Sweden; ^6^ West China Biomedical Big Data Center West China Hospital Sichuan University Chengdu China; ^7^ Med‐X Center for Informatics Sichuan University Chengdu China; ^8^ Center for Neurology Academic Specialist Center Stockholm Sweden

**Keywords:** amyotrophic lateral sclerosis, cohort study, disease progression, infection, nested case‐control study, risk factor

## Abstract

**Background:**

Infection has been suspected as a risk factor for amyotrophic lateral sclerosis (ALS). However, previous research has focused on specific pathogens and rarely examined the influence of infection on disease progression.

**Objectives:**

To assess whether hospital‐treated infections correlate with the risk and prognosis of ALS.

**Methods:**

Using data from the Swedish Motor Neuron Disease Quality Registry, we conducted three nested case‐control studies, including 1159 individuals diagnosed with ALS during 2015–2023 and 5795 age‐ and sex‐matched population controls, 1558 full‐sibling controls, and 680 spouse controls, respectively. We used conditional logistic regression to estimate the association of hospital‐treated infections with subsequent risk of ALS and Cox model to assess the association of pre‐ or post‐diagnostic infections with mortality after an ALS diagnosis.

**Results:**

Hospital‐treated infections before diagnosis were associated with an increased risk of ALS in the population comparison (odds ratio [OR] 1.31; 95% confidence interval [CI] 1.15–1.49). A similar association was noted after excluding infections within 3‐, 5‐, or 10‐years preceding ALS diagnosis and was confirmed in sibling and spouse comparisons, although results were not always statistically significant. Patients with a hospital‐treated infection before diagnosis were more likely to present with bulbar symptoms, poorer functional status, and higher prevalence of anxiety and depressive symptoms at diagnosis than others. Pre‐diagnostic infections were not associated with mortality, whereas post‐diagnostic infections were associated with increased mortality (hazard ratio [HR] 1.89; 95%CI 1.59–2.24) among ALS patients.

**Conclusion:**

Hospital‐treated infections are associated with an increased risk of ALS and may modify its clinical presentation at diagnosis. Post‐diagnostic infections are associated with poor survival in ALS.

## Graphical abstract

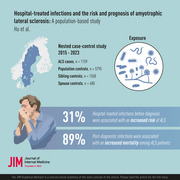

**Abbreviations**
ALS: amyotrophic lateral sclerosis
ALSFRS‐R: ALS Functional Rating Scale‐Revised
ATC: anatomical therapeutic chemical
BBB: blood–brain barrier
BMI: body mass index
CI: confidence interval
CNS: central nervous system
HAD: Hospital Anxiety and Depression
HR: hazard ratio
LISA: The longitudinal integrated database for health insurance and labor market studies
MND: motor neuron disease
MoCA: Montreal Cognitive Assessment
OR: odds ratio
SK‐ECAS: Edinburgh Cognitive and Behavioral ALS Screen

## Background

Amyotrophic lateral sclerosis (ALS) is the most prevalent form of motor neuron disease (MND), including 10%–15% of familial cases [1]. Although specific genetic mutations can be identified in approximately 70% of familial cases, such are only found in about 15% of sporadic cases [2]. Advanced age and male sex are the most established risk factors [3]. Body mass index (BMI) and educational level have also been proposed as risk factors; however, establishing a causal link is challenging [4]. Regardless, identification of potential risk factors, be it genetic or non‐genetic, can contribute toward a better understanding of disease mechanisms and help design preventive or therapeutic strategies.

Neuroinflammation is a typical pathological feature in ALS [5, 6]. Traditionally, the brain is considered immunologically privileged, safeguarded by the blood–brain barrier (BBB) and the blood‐cerebrospinal fluid barrier, which restricts the entry of immune cells and mediators to the central nervous system (CNS) [7, 8]. Certain pathogens, including bacteria, viruses, and fungi, can, however, penetrate these barriers and induce neuroinflammation [9, 10]. Furthermore, some pathogens can bypass the barriers through inter‐organ communication (e.g., the nose–brain axis) to directly access the CNS or release metabolites that traverse the BBB (e.g., via the lung–brain axis or gut–brain axis) [11]. Neuroinflammation might initiate as a defensive response to the invasion of pathogens, involving the activation of astrocytes and microglial cells, infiltration of peripheral immune cells, and production of proinflammatory cytokines [12, 13]. However, chronic neuroinflammation may also impede neurogenesis and lead to neuronal damage [14].

Despite the biological plausibility, research is relatively limited concerning a potential link between infection and ALS. The few existing studies often focused on specific pathogens (e.g., *Borrelia burgdorferi*, mycoplasmas, enteroviruses, and endogenous retroviruses) and used relatively small study materials with unknown representativeness of the source population [15, 16, 17, 18, 19, 20, 21]. Two other studies explored the association of hospital‐treated infections or use of antibiotics, as proxies for clinically evident infections, with the risk of ALS, with, however, little information on clinical characteristics of ALS [22, 23].

To this end, we utilized Swedish registers, including the Swedish MND Quality Registry, and conducted three nationwide nested case‐control studies to examine the association of hospital‐treated infections (and prescribed use of anti‐infectives) with the risk of ALS. In addition to age‐ and sex‐matched population controls, we used sibling and spouse controls to account for potential familial confounding. We also investigated the association of hospital‐treated infections with the clinical characteristics of ALS at the time of diagnosis and disease progression after diagnosis.

## Methods

### Data sources

The Swedish MND Quality Registry was established in 2015, encompassing approximately 85% of MND patients in Sweden and all MND patients in Stockholm [24]. The registry gathers a broad spectrum of clinical data and measurement results of biological samples at the time of diagnosis and thereafter. The present study was performed through linking the MND Quality Registry to several Swedish national population and health registers, including the Total Population Register, Multi‐Generation Register, Patient Register, Prescribed Drug Register, Causes of Death Register, Swedish Censuses, and “The longitudinal integrated database for health insurance and labor market studies” (LISA) [25], using the individually unique Swedish personal identification numbers. The Multi‐Generation Register includes largely complete information on familial links for individuals born since 1932 [26]. The Patient Register includes nationwide information on inpatient hospital visits since 1987 and over 80% of outpatient hospital visits since 2001 [27]. The Prescribed Drug Register includes nationwide information on prescribed use of medications since July 2005 [28]. LISA includes information on various socioeconomic variables such as educational level and household income [29].

### Study design

We included all patients (*N* = 1159) with newly diagnosed ALS in Sweden between January 2015 and July 2023, according to the MND Quality Registry. For this study, ALS was defined as cases meeting criteria for definite, probable, or possible ALS according to the revised El Escorial criteria (*N* = 1057) [30], as well as cases diagnosed with primary lateral sclerosis (PLS, *N* = 40) and progressive muscular atrophy (PMA, *N* = 62). According to the Gold Coast criteria, PMA is considered lower motor neuron‐predominant ALS, with often subclinical involvement of upper motor neurons [31]. We considered PLS as an ALS phenotype with almost exclusive upper motor neuron involvement [32].

First, we conducted three nested case‐control studies to evaluate the association between hospital‐treated infections and future risk of ALS, comparing the ALS patients to the population, sibling, and spouse controls, respectively. We used the sibling and spouse comparisons to assess potential familial confounding due to factors shared between siblings (e.g., genetic factors and early‐life environment and lifestyle) or spouses (i.e., adult‐life environment and lifestyle). Five population controls per case were randomly selected from the Total Population Register, using the method of incidence density sampling, and individually matched to the case by age and sex. Full siblings and spouses of the ALS patients were identified from the Multi‐Generation Register and the Total Population Register, respectively. The diagnosis date of each case was defined as the index date for both the case and its matched population and relative controls.

Second, to investigate the association between infections and clinical characteristics of ALS, we conducted a cohort study of the ALS patients with a follow‐up from the date of diagnosis until death, use of invasive ventilation, or August 31, 2023, whichever came first. Information on death was collected from the MND Quality Registry and the Causes of Death Register, whereas information on clinical characteristics, including initiation of invasive ventilation, was obtained from the MND Quality Registry. We used death or use of invasive ventilation, whichever came first, as the outcome of interest in the survival analysis.

Hospital‐treated infections were identified from the Patient Register for both the cases (before or after the index date) and their controls (before the index date), according to the ICD codes (Table S1). We studied any hospital‐treated infection as well as by site (CNS, gastrointestinal, skin, genitourinary, and respiratory) and type (bacterial, viral, or other) of infection [33]. As the Patient Register includes information on specialized care only, to ascertain infections attended by primary care, we additionally identified information on prescribed use of anti‐infectives through the Prescribed Drug Register, using the anatomical therapeutic chemical codes J01‐J05 and P01‐P03.

Clinical characteristics of the ALS patients were recorded in the MND Quality Registry at diagnosis and every 3 months thereafter, including onset site, family history, diagnostic delay, BMI, ALS Functional Rating Scale‐Revised (ALSFRS‐R), dyspnea (measured as a component of ALSFRS‐R and categorized as no dyspnea, occasional shortness of breath with exertion, shortness of breath on climbing stairs, shortness of breath on walking, shortness of breath at rest, or unknown), the Hospital Anxiety and Depression (HAD) scale, the Montreal Cognitive Assessment (MoCA), and Swedish Karolinska version of the Edinburgh Cognitive and Behavioral ALS Screen (SK‐ECAS) [24, 34]. We used information collected at the time of diagnosis (±90 days) to measure clinical presentation at diagnosis. Progression rate at diagnosis was calculated as (48—ALSFRS‐R score at diagnosis)/diagnostic delay (in months). To define the prevalence of anxiety and depressive symptoms at diagnosis, we employed revised HAD subscales and cut‐off values specific to MND patients, namely, ≥9 for anxiety and ≥8 for depressive symptoms [35].

Finally, we linked the cases and controls to LISA to identify information on educational attainment and household disposable income at the index date.

### Statistical analyses

#### Nested case‐control studies

We first described the characteristics of the cases and their respective controls. Second, as a descriptive analysis, we plotted the prevalence of hospital‐treated infections during all the 15 years preceding the index date for the cases and controls. Third, we used logistic regression conditioned on matching factors (i.e., age and sex in population comparison and family identifier in relative comparisons) to estimate odds ratios (ORs) with 95% confidence intervals (CIs) of ALS in relation to hospital‐treated infections. We separately analyzed hospital‐treated infections within 1 year, >1–3 years, >3–5 years, >5–10 years, and >10 years before the index date, using infections as a binary variable (yes or no). The choice of time windows followed a previous study [21] and because the median diagnostic delay was around 1 year for ALS patients in the present study. An individual could contribute to the analyses of multiple time windows in case of repeated events of infections.

To alleviate concern about potential reverse causation (e.g., due to diagnostic delay and pre‐clinical disease), we applied a lag time of 3 years in the subsequent analyses, that is, infections during the 3 years immediately preceding the index date were excluded. To explore possible cumulative effect of multiple infections, we counted repeated events of infections and categorized the total number of infections into groups of 0, 1, 2–3, or ≥4 events. In addition, we categorized hospital‐treated infections by severity (inpatient‐treated or outpatient‐treated), site, and type. To examine whether the results would differ for seasonal or epidemic infections, we performed a separate analysis for influenza (Table S1). To test whether the results would differ for ALS patients with or without a genetic cause, we also performed an analysis comparing ALS patients with or without *C9orf72* mutation to their respective controls. To assess the soundness of using a 3‐year lag time, in another two sensitivity analyses, we explored the use of different lag times (e.g., 5 or 10 years). Finally, we repeated these analyses for prescribed use of anti‐infectives alone (i.e., with no record in the Patient Register) as a proxy of milder infections not attended by specialized care.

In all analyses, we adjusted for educational attainment (<9 years, 9–12 years, >12 years, or unknown) and household disposable income (lowest 20%, middle, top 20%, or unknown), in addition to matching factors which were automatically controlled for. As siblings and spouses might differ in age and sex from their proband cases, age and sex were additionally adjusted for in the sibling and spouse comparisons.

#### Analysis on ALS patients

In the cohort analysis of patients with ALS, we first explored the association between previous hospital‐treated infections and clinical presentation of ALS at diagnosis. We first used univariate analysis to compare clinical characteristics between patients with versus without pre‐diagnostic infections, using *t*‐test for continuous variables and Chi‐square test for binary variables. We then performed multivariable analysis, using logistic regression with adjustment for age, sex, educational attainment, household disposable income, and site of onset, to assess the association of pre‐diagnostic infections with the odds of bulbar onset, family history, lower ALSFRS‐R (<37.6), longer diagnostic delay (≥12.4 months), greater progression rate (≥1.1), higher BMI (≥23.2), anxiety and depressive symptoms, lower MoCA (<26), and lower SK‐ECAS (ALS specific score <82 and total score <108). Missing values in the covariates were categorized as unknown. The cut‐off values for ALSFRS‐R, diagnostic delay, progression rate, and BMI were defined using the mean values of the entire cohort. The cut‐off value for MoCA was defined according to the suggested cut‐off for MoCA, whereas the cut‐off values of SK‐ECAS were defined according to our previous study [34].

Finally, we assessed the association between hospital‐treated infections and the risk of death (or use of invasive ventilation) following ALS diagnosis, using Cox model with adjustment for age, sex, site of onset, diagnostic delay, ALSFRS‐R score at diagnosis, BMI at diagnosis, dyspnea, educational attainment, and household disposable income. Time since diagnosis was used as the time scale. We separately analyzed pre‐diagnostic and post‐diagnostic infections. Pre‐diagnostic infections were treated as binary exposure (yes or no). For post‐diagnostic infections, patients were followed as unexposed until their first post‐diagnostic infection, at which point they were switched to the exposed group. A robust sandwich estimator was used to address clustered residuals between repeated events. We also conducted a sensitivity analysis using death as the sole survival outcome, irrespective of the use of invasive ventilation, as patients with infections may be more likely to receive invasive ventilation.

All analyses were conducted in Python (version 3.9) and R software (version 4.3). A 2‐sided *p* value <0.05 was considered statistically significant.

## Results

### Nested case‐control studies

The population comparison included 1159 cases and 5795 controls; the corresponding numbers were 780 and 1558 for sibling comparison and 680 and 680 for spouse comparison (Table 1). The mean age at ALS diagnosis was 67 years, and there were slightly more males than females among the cases.

**Table 1:** Characteristics of the patients with amyotrophic lateral sclerosis (ALS) and their population and relative controls.

	Population analysis		Sibling analysis		Spouse analysis	
Characteristics	Cases (*N* = 1159)	Controls (*N* = 5795)	Cases (*N* = 780)	Controls (*N* = 1558)	Cases (*N* = 680)	Controls (*N* = 680)
Age, mean (SD)	67.3 (11.6)	67.3 (11.6)	66.4 (11.1)	66.4 (12.2)	67.8 (11.2)	66.8 (12.1)
Sex, *N* (%)						
Male	633 (54.6)	3165 (54.6)	439 (56.3)	772 (49.6)	390 (57.4)	291 (42.8)
Female	526 (45.4)	2630 (45.4)	341 (43.7)	786 (50.4)	290 (42.6)	389 (57.2)
Educational attainment, *N* (%)						
<9 years	240 (20.7)	1393 (24.0)	139 (17.8)	241 (15.5)	134 (19.7)	111 (16.3)
9–12 years	466 (40.2)	2509 (43.3)	321 (41.2)	587 (37.7)	275 (40.4)	291 (42.8)
>12 years	436 (37.6)	1826 (31.5)	310 (39.7)	483 (31.0)	264 (38.8)	275 (40.4)
Unknown	17 (1.5)	67 (1.2)	10 (1.3)	247 (15.9)	7 (1.0)	3 (0.4)
Household disposable income, *N* (%)						
Lowest 20%	214 (18.5)	1336 (23.1)	125 (16.0)	254 (16.3)	25 (3.7)	48 (7.1)
Middle	681 (58.8)	3478 (60.0)	453 (58.1)	783 (50.3)	443 (65.1)	424 (62.4)
Top 20%	255 (22.0)	978 (16.9)	194 (24.9)	280 (18.0)	212 (31.2)	208 (30.6)
Unknown	9 (0.8)	3 (0.1)	8 (1.0)	241 (15.5)	–	–

Abbreviation: SD, standard deviation.

**Fig. 1:**
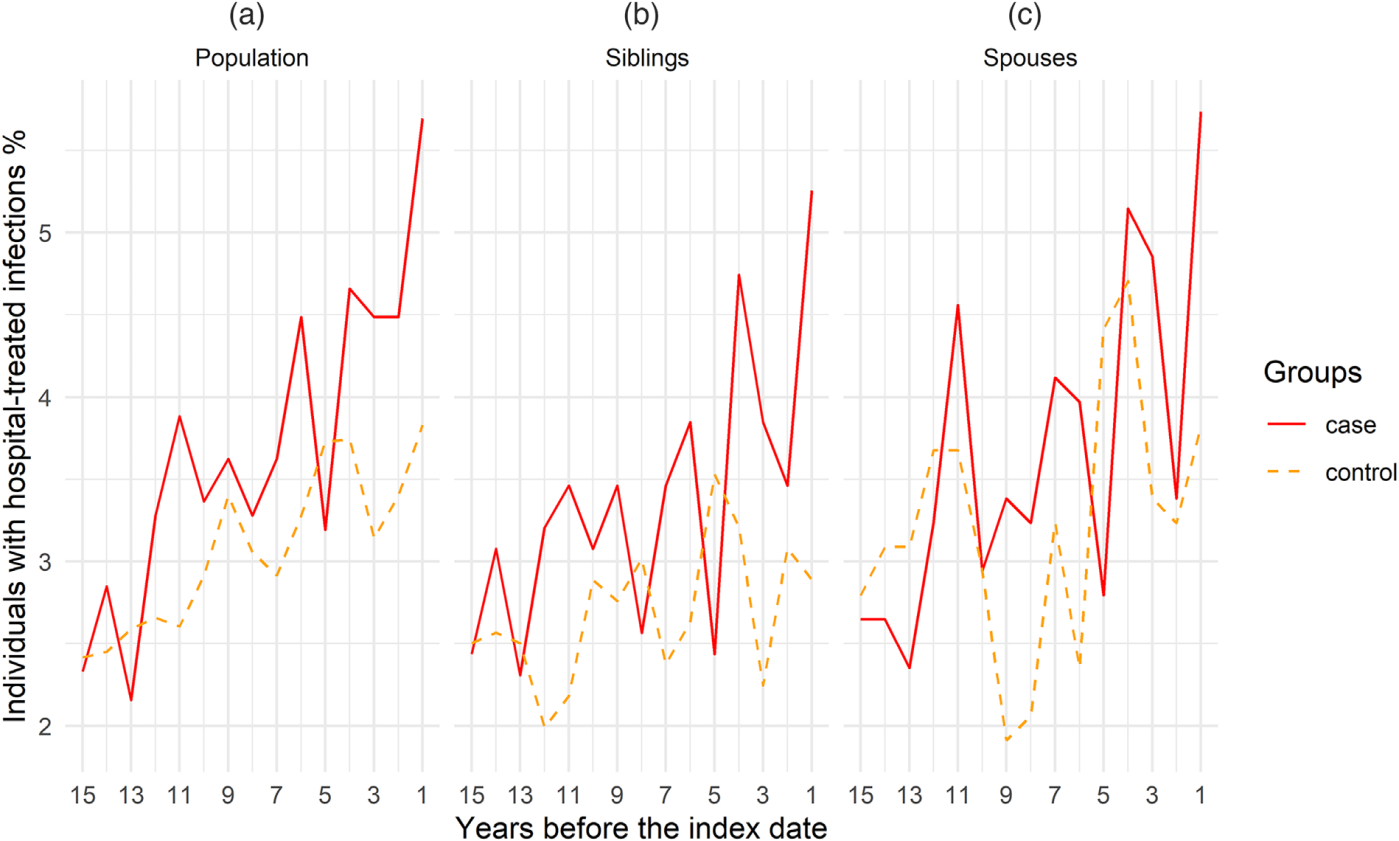
Prevalence of hospital‐treated infections during the 15 years before the index date among patients with amyotrophic lateral sclerosis (ALS) and their controls: (a) population controls, (b) sibling controls, and (c) spouse controls. The x‐axis shows the individual years before the index date (e.g., “1” = “1st year before the index date”). The different estimates of prevalence observed in cases of different comparisons were due to the fact that only cases with a sibling or spouse control were included in the sibling or spouse comparison.

Fig. 1 illustrates the percentage of individuals who had a hospital‐treated infection during a specific year before the index date. Overall, we observed a slightly higher prevalence of hospital‐treated infections during the 15 years before the index date among cases compared to different control groups. In the population comparison, the multivariable‐adjusted OR of ALS was 1.31 (95%CI 1.15–1.49) for infections experienced any time before index date, 1.52 (95%CI 1.14–2.02) for infections experienced during the year before index date, and 1.39 (95%CI 1.09–1.78) for infections experienced during >1–3 years before index date (Table 2). No association was noted for infections experienced >3–5 years before index date (OR 1.02; 95%CI 0.79–1.31); however, a positive association was again noted for infections experienced during >5–10 years (OR 1.19; 95%CI 0.99–1.43) and >10 years (OR 1.19; 95%CI 1.03–1.37) before index date. Consistent effect sizes were observed in the sibling and spouse comparisons, although the associations in sibling comparisons were not statistically significant. The analysis of prescribed use of anti‐infectives rendered similar results (Table S2).

**Table 2:** Previous hospital‐treated infections and risk of amyotrophic lateral sclerosis (ALS)—Analysis by the timing of infection.

	Population analysis			Sibling analysis			Spouse analysis		
Groups	Cases (exposed, %)	Controls (exposed, %)	OR (95%CI)^a^	Cases (exposed, %)	Controls (exposed, %)	OR (95%CI)^a^	Cases (exposed, %)	Controls (exposed, %)	OR (95%CI)^a^
**Any time before diagnosis**	546 (47.1)	2364 (40.8)	**1.31 (1.15–1.49)**	359 (46.0)	634 (40.7)	1.16 (0.96–1.42)	333 (49.0)	287 (42.2)	**1.43 (1.13–1.81**)
**Years before diagnosis**									
<1 year	66 (5.7)	222 (3.8)	**1.52 (1.14–2.02)**	41 (5.3)	45 (2.9)	1.54 (0.97–2.45)	39 (5.7)	26 (3.8)	1.35 (0.79–2.31)
>1–3 years	92 (7.9)	349 (6.0)	**1.39 (1.09–1.78)**	53 (6.8)	74 (4.7)	1.22 (0.83–1.79)	49 (7.2)	42 (6.2)	1.19 (0.76–1.85)
>3–5 years	78 (6.7)	388 (6.7)	1.02 (0.79–1.31)	51 (6.5)	92 (5.9)	0.89 (0.61–1.30)	49 (7.2)	52 (7.6)	0.95 (0.62–1.46)
>5–10 years	164 (14.2)	704 (12.1)	1.19 (0.99–1.43)	101 (12.9)	169 (10.8)	1.19 (0.89–1.58)	97 (14.3)	69 (10.1)	**1.46 (1.04–2.05)**
>10 years	355 (30.6)	1571 (27.1)	**1.19 (1.03–1.37)**	240 (30.8)	443 (28.4)	1.14 (0.92–1.41)	220 (32.4)	196 (28.8)	**1.29 (1.01–1.64)**

Abbreviations: CI, confidence interval; OR, odds ratio.

^a^ Derived from logistic regression, conditioned on age‐ and sex‐matched pairs, sibling pairs, or spouse pairs, respectively, after adjustment for educational attainment and household disposable income in all analyses, and additionally age and sex in the sibling and spouse analyses.

Bold values are statistically significant *p* < 0.05.

In the subsequent analyses, we focused on the population comparison with a lag time of 3 years. We observed an increasing risk of ALS in relation to an increasing number of anti‐infective use but not to the number of hospital‐treated infections (Table S3). The analysis of infections by severity, site, and type did not reveal a specific result pattern, except that a statistically significant association was noted for outpatient‐ rather than inpatient‐treated infections (Table S4). Influenza also appeared to be associated with a higher risk of ALS, but the result was not statistically significant (OR 1.46; 95%CI 0.72–2.96). Among the 436 ALS patients with information on genetic cause, 41 had the *C9orf72* mutation. The result did not differ clearly by the status of *C9orf72* mutation (OR 1.27; 95%CI 0.63–2.58 for ALS with *C9orf72* and OR 1.33; 95%CI 1.06–1.67 for ALS without *C9orf72*). Finally, using a lag time of 5 or 10 years yielded very similar results as the use of a 3‐year lag time (Table S5).

### Analysis on ALS patients

In the univariate analysis, patients with a hospital‐treated infection before ALS diagnosis were more likely to have bulbar onset (*p* = 0.04), a higher progression rate (*p* = 0.01), as well as anxiety (*p* = 0.02) and depressive (*p* = 0.02) symptoms, compared to other patients, at the time of diagnosis (Table S6). After multivariable adjustment, having an event of hospital‐treated infection prior to diagnosis was associated with higher odds of bulbar onset (OR 1.38; 95%CI 1.00–1.91), lower ALSFRS‐R (OR 1.45; 95%CI 1.02–2.06), anxiety symptoms (OR 2.05; 95%CI 1.08–3.91), and depressive symptoms (OR 2.33; 95%CI 1.08–5.06) at the time of diagnosis (Table 3).

**Table 3:** Previous hospital‐treated infections and clinical presentation of amyotrophic lateral sclerosis (ALS) at the time of diagnosis.

Clinical characteristics	Yes (infected/total; %)	No (infected/total; %)	OR (95%CI)^a^
Onset site: bulbar	129/237 (54.4)	208/470 (44.3)	**1.38 (1.00–1.91)**
Family history: yes	86/178 (48.3)	460/981 (46.9)	1.03 (0.73–1.46)
ALSFRS‐R at diagnosis: <37.6	158/302 (52.3)	126/284 (44.4)	**1.45 (1.02–2.06)**
Diagnostic delay in months: ≥12.4	140/283 (49.5)	135/282 (47.9)	1.03 (0.73–1.44)
Progression rate at diagnosis: ≥ 1.1	90/170 (52.9)	185/395 (46.8)	1.30 (0.89–1.89)
BMI at diagnosis: ≥23.2	162/328 (49.4)	162/328 (49.4)	1.03 (0.75–1.42)
Anxiety: yes	36/54 (66.7)	105/222 (47.3)	**2.05 (1.08–3.91)**
Depression: yes	26/37 (70.3)	115/239 (48.1)	**2.33 (1.08–5.06)**
MoCA: <26	36/72 (50.0)	77/161 (47.8)	1.30 (0.71–2.40)
SK‐ECAS ALS specific: <82	46/90 (51.1)	53/100 (53.0)	1.05 (0.56–1.98)
SK‐ECAS total: <108	41/84 (48.8)	58/106 (54.7)	0.94 (0.49–1.82)

Abbreviations: ALSFRS‐R, ALS functional rating scale‐revised; BMI, body mass index; CI, confidence interval; MoCA, Montreal Cognitive Assessment; OR, odds ratio; SK‐ECAS, Edinburgh Cognitive and Behavioral ALS Screen.

^a^ Derived from logistic regression, with adjustment for age at diagnosis, sex, educational attainment, and household disposable income in all analyses and additionally onset site for the other clinical characteristics.

Bold values are statistically significant *p* < 0.05.

Finally, each event of hospital‐treated infection after ALS diagnosis was associated with a nearly doubled risk of death (hazard ratio [HR] 1.89; 95%CI 1.59–2.24) (Table 4). Infections experienced before diagnosis were not associated with mortality risk, however (HR 1.01; 95%CI 0.88–1.16). In the sensitivity analysis excluding the use of invasive ventilation as a survival outcome, we observed consistent results for post‐diagnostic infections (HR 2.15; 95%CI 1.80–2.57).

**Table 4:** Hospital‐treated infections before or after diagnosis and risk of death among patients with amyotrophic lateral sclerosis (ALS).

Timing of infection	*N* of deaths/person‐years (IR per 1000 person‐years) among unexposed patients	*N* of deaths/person‐years (IR per 1000 person‐years) among exposed patients	HR (95%CI)^a^
Any time after ALS diagnosis^b^	680/1800 (377.78)	190/242 (785.12)	**1.95 (1.64–2.31)**
Any time before ALS diagnosis	462/1105 (418.10)	408/916 (445.41)	1.01 (0.88–1.16)

Abbreviations: CI, confidence interval; HR, hazard ratio; IR, incidence rate.

^a^ Derived from Cox model, adjusted for age at diagnosis, sex, onset site, diagnostic delay, ALSFRS‐R at diagnosis, body mass index at diagnosis, dyspnea, educational attainment, and household disposable income. Time since diagnosis was used as time scale.

^b^ Robust sandwich estimator was applied to account for relatedness between repeated events of infections among patients who experienced hospital‐treated infections after receiving a diagnosis of ALS.

Bold values are statistically significant *p* < 0.05.

## Discussion

To the best of our knowledge, this is the first nationwide study with virtually complete follow‐up to examine the role of clinically evident infections, including infections requiring specialized care (inpatient or outpatient) and infections attended by primary care, on the risk and prognosis of ALS. Our main finding is that previous hospital‐treated infections were associated with an increased subsequent risk of ALS, and that this association remained robust when applying a lag‐time of 3, 5, or 10 years before ALS diagnosis, which alleviated the concern of potential reverse causation. The use of multivariable adjustment and the similarity in results obtained by comparing ALS patients with the population and relative controls argue against confounding as an important explanation for this finding. Our study also indicates that infections experienced before diagnosis may correlate with the clinical presentation of ALS at diagnosis, including site of onset, functional status, and presence of anxiety and depressive symptoms. Further, after controlling for clinical characteristics at diagnosis, we found that an infection experienced after ALS diagnosis was associated with an almost doubled mortality risk. Collectively, our findings provide novel evidence indicating that clinically evident infections may play a role in the pathogenesis of ALS long before its clinical onset and are important in the clinical management of ALS patients.

We did not demonstrate an association between hospital‐treated infections (identified through specialized care) and risk of ALS in a previous study during 1970–2016 [22]. As the Swedish Patient Register included data on outpatient care since 2001 only, the majority of infections identified in this previous study were treated in inpatient care. In contrast, the present study had a study period of 2015–2023, including largely complete data on outpatient‐treated infections for over 15 years for most participants. In fact, in the present study, we observed an association for outpatient‐ but not inpatient‐treated infections. One possible explanation for this discrepancy is the lower number of inpatient‐treated infections and the resultant limited statistical power to detect an association with mild to moderate magnitude. Another possibility is that patients with inpatient‐treated infections may have received different treatments than those with outpatient‐treated infections, for example, intravenous use of anti‐infectives or a wider spectrum of antibiotics. Regardless, the positive association for prescribed use of anti‐infectives noted in the present study is consistent with another study of our group on antibiotics use [23]. Finally, the increasing risk of ALS noted in relation to the increasing number of anti‐infective use adds additional evidence for a potentially causal link between infections and ALS, although the trend is less clear for hospital‐treated infections.

Although electromyography findings suggest that rapid cell loss and death of motor neurons occur just before the clinical onset of ALS, alterations in the function of motor neurons may debut much earlier [36, 37, 38]. Indeed, among patients with familial ALS, it has been shown that the level of neurofilament light may increase well before any clinical sign is evident [39]. Rodent models further indicate that morphometric and physiological abnormalities can present as early as the prenatal age, whereas gait alterations and muscle weakness could manifest 1 month postnatal [38]. In the present study, we found a positive association between infections experienced as early as >10 years before diagnosis and a higher risk of ALS. Although a positive association was observed also for infections experienced during 1 or 3 years before ALS diagnosis, this finding might be a result of reverse causality, considering diagnostic delay and pre‐clinical disease [40, 41].

A multistep process has been hypothesed [42, 43], suggesting that approximately six cumulative steps are necessary to initiate the disease process of sporadic ALS [42]. The finding of our study suggests that infection and its subsequent pathological changes (such as inflammatory responses and neuroinflammation) could potentially represent one such step. Although the underlying mechanisms of this association remain unclear, several biological studies have provided plausible explanations. Chronic intraperitoneal injection of lipopolysaccharide (LPS), which simulates systemic bacterial infection in mice, has been shown to promote TDP‐43 mislocalization, a pathological feature observed in approximately 95% of ALS cases [44]. Moreover, interferon‐gamma, typically upregulated following infection, has also been implicated in promoting TDP‐43 aggregation [45]. Elevated levels of TDP‐43 have demonstrated the potential to interact with LPS, further enhancing the production of inflammatory cytokines in neuronal and glial cells [46]. Another possibility is that infections may interact with the microbiome and induce dysbiosis in the gut [47]. This could increase intestinal permeability, allowing bacterial products, including endotoxins, to translocate into the systemic circulation and potentially trigger systemic neuroinflammation and brain microglial activation [48, 49]. In addition, a dysbiotic microbiota may reduce the production of beneficial short‐chain fatty acids, such as butyrate, which are considered to have neuroprotective effects [50]. Regardless, as a positive association has also been suggested between hospital‐treated infections and risk of other neurodegenerative diseases, for example, Alzheimer's disease and Parkinson's disease [22], it is unlikely that the proposed mechanisms are specific to motor neurons or ALS.

Interestingly, we found pre‐diagnostic infections to be correlated with some aspects of clinical presentation of ALS at diagnosis, namely, site of onset, functional status, and anxiety and depressive symptoms. However, as this analysis is exploratory in nature, these findings may be partly due to false positives given the relatively limited statistical power and multiple testing. Although whether infections indeed modulate heterogeneity of ALS needs to be studied further, the increased mortality in relation to infections following ALS diagnosis suggests the importance of preventing and managing infections in patients with ALS. Infections may exacerbate neuroinflammation, resulting in accelerated neuronal death, or systemic inflammation, leading to risk of death in general [51, 52, 53]. It is, however, unlikely that the infection‐related increase in mortality is specific to ALS, as infections have similarly been shown to increase risk of mortality among patients with other neurodegenerative diseases [54].

The main strength of the study is the use of the Swedish MND Quality Registry with the inclusion of only incident cases, detailed information on clinical characteristics at the time of diagnosis and during follow‐up, and the use of the Swedish population registers to identify three control groups. This approach ensured that we included the majority of ALS patients diagnosed during the study period from the entire country, a truly representative sample of population controls, and all eligible sibling and spouse controls. The use of relative controls provided the possibility to adjust for familial confounding, in addition to multivariable adjustment. Another strength is the use of Swedish health registers to identify infections requiring treatment, with information on the severity, type, and site of the infection, both before and after ALS diagnosis. Our study also has limitations. First, although sibling and spouse comparisons adjusted for unknown or unmeasured factors shared between family members (e.g., genetic factors, lifestyle, and residential area), residual confounding due to factors not shared between siblings and spouses may still exist. Second, although we had a relatively large sample size, some subgroup analyses (e.g., analysis on time of hospital‐treated infections in sibling comparison) still had limited statistical power, leading to concern of false negative findings. Interpretation of these analyses should therefore be done with caution. Finally, given that the incomplete coverage of the Swedish MND Quality Registry is primarily due to administrative reasons (e.g., not all clinics treating MND patients have reported their patients to the Registry), we speculate a relatively minor impact of such on the study results given that the Registry includes approximately 85% of all MND patients in the country.

In conclusion, hospital‐treated infections, identified through specialized care or prescribed use of anti‐infectives, were associated with an increased future risk of ALS. Pre‐diagnostic infections were also correlated with clinical presentation of ALS at diagnosis, namely site of onset, functional status, and anxiety and depressive symptoms, whereas infections experienced after diagnosis were associated with an increased mortality risk among ALS patients.

## Author contributions

Fang Fang and Yihan Hu contributed to the conception and design of the study. Charilaos Chourpiliadis, Fang Fang, Caroline Ingre, Yudi Pawitan, Viktor H. Ahlqvist, and Yihan Hu contributed to acquisition and analysis of data. Yihan Hu, Huan Song, Fredrik Piehl, Caroline Ingre, Jiangwei Sun, Viktor H. Ahlqvist and Fang Fang contributed to drafting the text or preparing the Figs.

## Conflict of interest statement

The authors declare no conflicts of interest.

## Disclosure

Caroline Ingre has consulted for Cytokinetics, Pfizer, BioArctic, Novartis, Tikomed, Ferrer, Amylyx, Prilenia, and Mitsubishi. She is also a board member of Tobii Dynavox; all outside the submitted work.

## Ethics statement

Ethical approval for the study was obtained from the Swedish Ethical Review Authority (DNR: 2022‐02314‐01).

## Supporting information




**Supplementary Table 1**: ICD codes used for the identification of hospital‐treated infections.
**Supplementary Table 2**: Use of anti‐infectives and risk of amyotrophic lateral sclerosis (ALS).
**Supplementary Table 3**: Number of hospital‐treated infections (or anti‐infective use) and risk of amyotrophic lateral sclerosis (ALS) analysis with a lag time of 3 years.
**Supplementary Table 4**: Previous hospital‐treated infections and risk of amyotrophic lateral sclerosis (ALS)‐analysis by severity, site, and type of infection using a lag time of 3 years.
**Supplementary Table 5**: Previous hospital‐treated infections and risk of amyotrophic lateral sclerosis (ALS) analysis with various lag times.
**Supplementary Table 6**: Previous hospital‐treated infections and presentation of amyotrophic lateral sclerosis (ALS) at the time of diagnosis univariate analysis.

## Data Availability

The data used in the present study are not publicly available due to Swedish regulations and GDPR. Please contact the Swedish Motor Neuron Disease Quality Registry (https://www.neuroreg.se/) or the corresponding author for more information about data access.
