# Supplementary material for: Hospital‐treated infections and the risk and prognosis of amyotrophic lateral sclerosis: A population‐based study
Source: J Intern Med. 2025 Aug 5;298(4):349–60. doi: 10.1111/joim.70008 (PMC12459329; doi:10.1111/joim.70008)
Supplement: Supplementary file 1 — Supplementary Table 1: ICD codes used for the identification of hospital‐treated infections. Supplementary Table 2: Use of anti‐infectives and risk of amyotrophic lateral sclerosis (ALS). Supplementary Table 3: Number of hospital‐treated infections (or anti‐infective use) and risk of amyotrophic lateral sclerosis (ALS) analysis with a lag time of 3 years. Supplementary Table 4: Previous hospital‐treated infections and risk of amyotrophic lateral sclerosis (ALS)‐analysis by severity, site, and type of infection using a lag time of 3 years. Supplementary Table 5: Previous hospital‐treated infections and risk of amyotrophic lateral sclerosis (ALS) analysis with various lag times. Supplementary Table 6: Previous hospital‐treated infections and presentation of amyotrophic lateral sclerosis (ALS) at the time of diagnosis univariate analysis. [file JOIM-298-349-s001.docx]

Supplementary Table 1. ICD codes used for the identification of hospital-treated infections

| Infection | ICD version | ICD codes |
| --- | --- | --- |
| *Site of infection* |  |  |
| Central nervous system | 8 | 013,062,063,064,065,066,071,094,292,320,323,324,390,474,040-046 |
|  | 9 | 013,062,063,064,071,094,320,323,326,392,045-049 |
|  | 10 | A17,A80-A89,G00,G01,G02,G04,G05,I02 |
| Gastrointestinal | 8 | 014,123,127,129,540,567,000-009 |
|  | 9 | 014,123,127,129,540,567,001-009 |
|  | 10 | A00-A09,B71,B81,B82,K35,K65,K67 |
| Skin | 8 | 110,111,050-057,680-686 |
|  | 9 | 110,111,050-057,680-686 |
|  | 10 | B00-B09,B35,B36,B43,L00-L08 |
| Genitourinary | 8 | 016,590,595,597 |
|  | 9 | 016,590,595,597 |
|  | 10 | N30,N34,O23 |
| Respiratory | 8 | 010,011,012,033,034,075,115,116,490,501,503,510,460-466,470-474,480-486 |
|  | 9 | 010,011,012,033,034,075,115,116,473,475,487,490,510, 460-466,480-486 |
|  | 10 | A15,A16,A37,A38,B27,B39,B40,B41,B42,B44,B59,J00-J06,J10,J12-J18,J20-J22,J32,J36,J40-J42,J86,P23 |
| *Type of infection* |  |  |
| Bacterial | 8 | 595,597,612,613,614,616,620,622,630,635,670,678,680,000,001,002,003,004,005,073,076,080,081,082,083,320,362,  380,381,382,383,421,461,481,482,501,510,567,590,681,682,684,710,720,010-019,020-027,030-039,090-099,100-104,390-392 |
|  | 9 | 001,002,003,004,005,073,076,077,078,079,080,081,082,083,320,381,382,383,383,421,461,475,481,482,510,567,590,595,597,670,730,010-018,020-027,030-041,090-099,100-104,390-392,614-616,680-686 |
|  | 10 | A00,A01,A02,A03,A04,A05,A15-A19,A20-A28,A30-A49,A50-A58,A65-A69,A70-A74,A75-A79,B95,B96,  G00,G01,H60,H70,I00-I02,I33,J01,J13,J14,J15,J36,J86,K65,L00-L08,M00,M86,N30,N34,N70-N77,O23,O85,O86,  P36 |
| Viral | 8 | 075,360,420,422,460,464,465,466,480,040-046,050-057,060-068,070-079,470-474 |
|  | 9 | 070,071,072,074,075,077,078,079,372,420,422,460,464,465,466,480,487,647,711,045-049,050-057,060-066 |
|  | 10 | A08,A60,A80-A89,A90-A99,B00-B09,B15-B19,B20-B24,B25-B34,B27,B97,B99,H10,I30,I40,J00,J04, J05,J06,J10,J12,J20,J21,O98,P35,Z21 |
| Other | 8 | 006,007,008,009,084,085,086,087,088,089,110-117,120-129,130-136,292,323,324,363,462,463,483,484,485,486,  490,503,540,610,611,615,683,685,686,732,763,Y41 |
|  | 9 | 006,007,008,009,084,085,086,087,088,110-118,120-129, 130-136,137-139,323,326,370,462,463,473,483,484,485,  486,490,540,675,771 |
|  | 10 | A06,A07,A09,A59,A63,A64,B35-B49,B50-B64,B65-B83,B85-B89,B90-B94,G02,G04,G05,H16,H32,J02,J03,J16,  J17,J18,J22,J32,J40,J41,J42,K35,K67,M01,M02,M03,O91,P23,P37,P38,P39,V02,Z22 |
| *Specific infection* |  |  |
| Influenza | 8 | 470-474 |
|  | 9 | 487 |
|  | 10 | J09-J11 |

Supplementary Table 2. Use of anti-infectives and risk of amyotrophic lateral sclerosis (ALS)

| **Years before diagnosis** | **Cases (exposed, %)** | **Controls (exposed, %)** | **OR (95% CI) ^a^** |
| --- | --- | --- | --- |
| <1 year | 307 (26.5) | 1257 (21.7) | **1.29 (1.12-1.50)** |
| >1-3 years | 421 (36.3) | 1972 (34.0) | 1.09 (0.96-1.25) |
| >3-5 years | 434 (37.4) | 2076 (35.8) | 1.06 (0.93-1.21) |
| >5-10 year | 759 (65.5) | 3407 (58.8) | **1.32 (1.16-1.51)** |
| >10 years | 652 (56.3) | 2945 (50.8) | **1.29 (1.12-1.49)** |

OR: odds ratio; CI: confidence interval.

**^a^**Derived from logistic regression, conditioned on age- and sex-matched pairs, after adjustment for educational attainment and household disposable income.

Supplementary Table 3. Number of hospital-treated infections (or anti-infective use) and risk of amyotrophic lateral sclerosis (ALS) – analysis with a lag time of 3 years

|  |  | **Population analysis** | | |
| --- | --- | --- | --- | --- |
| **Exposure** | **Number** | **Cases (exposed, %)** | **Controls (exposed, %)** | **OR (95% CI) ^a^** |
| Hospital-treated infections | 0 | 680 (58.7) | 3646 (62.9) | Ref |
|  | 1 | 282 (24.3) | 1279 (22.1) | **1.19 (1.02-1.39)** |
|  | 2-3 | 151 (13.0) | 645 (11.1) | **1.26 (1.03-1.53)** |
|  | ≥4 | 46 (4.0) | 225 (3.9) | 1.09 (0.78-1.52) |
| Use of anti-infectives | 0 | 181 (15.6) | 1186 (20.5) | Ref |
|  | 1 | 206 (17.8) | 1141 (19.7) | 1.20 (0.96-1.49) |
|  | 2-3 | 320 (27.6) | 1539 (26.6) | **1.36 (1.11-1.66)** |
|  | ≥4 | 452 (39.0) | 1929 (33.3) | **1.54 (1.27-1.88)** |

OR: odds ratio; CI: confidence interval.

**^a^**Derived from logistic regression, conditioned on age- and sex-matched pairs, after adjustment for educational attainment and household disposable income.

Supplementary Table 4. Previous hospital-treated infections and risk of amyotrophic lateral sclerosis (ALS) - analysis by severity, site, and type of infection using a lag time of 3 years

|  | **Population analysis** | | |
| --- | --- | --- | --- |
| **Infection** | **Cases (exposed, %)** | **Controls (exposed, %)** | **OR (95% CI) ^a^** |
| **Severity of infection** |  |  |  |
| Inpatient-treated | 278 (24.0) | 1329 (22.9) | 1.08 (0.93-1.25) |
| Outpatient-treated | 314 (27.1) | 1318 (22.7) | **1.25 (1.08-1.44)** |
| **Site of infection** |  |  |  |
| CNS | 6 (0.5) | 52 (0.9) | 0.57 (0.24-1.35) |
| Gastrointestinal | 96 (8.3) | 464 (8.0) | 1.04 (0.82-1.30) |
| Skin | 68 (5.9) | 319 (5.5) | 1.07 (0.82-1.41) |
| Genitourinary | 34 (2.9) | 138 (2.4) | 1.25 (0.85-1.83) |
| Respiratory | 174 (15.0) | 727 (12.5) | **1.23 (1.03-1.48)** |
| **Type of infection** |  |  |  |
| Bacterial | 249 (21.5) | 1124 (19.4) | 1.15 (0.98-1.35) |
| Viral | 156 (13.5) | 683 (11.8) | 1.18 (0.98-1.42) |
| Other | 80 (6.9) | 293 (5.1) | **1.36 (1.05-1.76)** |
| **Specific infection** |  |  |  |
| Influenza | 10 (0.9) | 37 (0.6) | 1.46 (0.72-2.96) |

OR: odds ratio; CI: confidence interval; CNS: central nervous system.

**^a^**Derived from logistic regression, conditioned on age- and sex-matched pairs, after adjustment for educational attainment and household disposable income.

Supplementary Table 5. Previous hospital-treated infections and risk of amyotrophic lateral sclerosis (ALS) – analysis with various lag times

|  |  | **Population analysis** | | | **Sibling analysis** | | | **Spouse analysis** | | |
| --- | --- | --- | --- | --- | --- | --- | --- | --- | --- | --- |
| **Exposure** | **Lag time** | **Cases (exposed, %)** | **Controls (exposed, %)** | **OR (95% CI) ^a^** | **Cases (exposed, %)** | **Controls (exposed, %)** | **OR (95% CI) ^a^** | **Cases (exposed, %)** | **Controls (exposed, %)** | **OR (95% CI) ^a^** |
| Any hospital-treated infection | 0 year | 546 (47.1) | 2364 (40.8) | **1.31 (1.15-1.49)** | 359 (46.0) | 634 (40.7) | 1.16 (0.96-1.42) | 333 (49.0) | 287 (42.2) | **1.43 (1.13-1.81)** |
|  | 3 years | 479 (41.3) | 2149 (37.1) | **1.20 (1.05-1.37)** | 317 (40.6) | 578 (37.1) | 1.13 (0.92-1.38) | 294 (43.2) | 256 (37.6) | **1.39 (1.10-1.76)** |
|  | 5 years | 446 (38.5) | 19815 (34.2) | **1.21 (1.06-1.38)** | 293 (37.6) | 539/ (34.6) | 1.15 (0.94-1.40) | 272 (40.0) | 233 (34.3) | **1.38 (1.09-1.75)** |
|  | 10 years | 355 (30.6) | 1571 (27.1) | **1.19 (1.03-1.37)** | 240 (30.8) | 443 (28.4) | 1.14 (0.92-1.41) | 220 (32.4) | 196 (28.8) | **1.29 (1.01-1.64)** |
| Inpatient-treated infection | 0 year | 307 (26.5) | 1429 (24.7) | 1.12 (0.97-1.30) | 199 (25.5) | 390 (25.0) | 1.04 (0.83-1.30) | 188 (27.6) | 169 (24.9) | 1.21 (0.93-1.56) |
|  | 3 years | 278 (24.0) | 1329 (22.9) | 1.08 (0.93-1.25) | 185 (23.7) | 361 (23.2) | 1.07 (0.85-1.34) | 167 (24.6) | 160 (23.5) | 1.13 (0.87-1.48) |
|  | 5 years | 265 (22.9) | 1235 (21.3) | 1.11 (0.95-1.29) | 178 (22.8) | 341 (21.9) | 1.13 (0.90-1.42) | 159 (23.4) | 151 (22.2) | 1.16 (0.89-1.52) |
|  | 10 years | 234 (20.2) | 1074 (18.5) | 1.12 (0.96-1.32) | 160 (20.5) | 311 (20.0) | 1.12 (0.88-1.43) | 137 (20.1) | 142 (20.9) | 1.02 (0.77-1.35) |
| Outpatient-treated infection | 0 year | 383 (33.0) | 1549 (26.7) | **1.35 (1.17-1.55)** | 249 (31.9) | 382 (24.5) | **1.34 (1.08-1.65)** | 233 (34.3) | 189 (27.8) | **1.49 (1.15-1.92)** |
|  | 3 years | 314 (27.1) | 1318 (22.7) | **1.25 (1.08-1.44)** | 205 (26.3) | 333 (21.4) | 1.24 (0.99-1.54) | **196 (28.8)** | **159 (23.4)** | **1.46 (1.12-1.90)** |
|  | 5 years | 278 (24.0) | 11565 (19.9) | **1.25 (1.07-1.45)** | 178 (22.8) | 291 (18.7) | 1.25 (0.98-1.58) | **173 (25.4)** | **133 (19.6)** | **1.52 (1.15-2.02)** |
|  | 10 years | 180 (15.5) | 751 (13.0) | **1.22 (1.02-1.46)** | 118 (15.1) | 189 (12.1) | 1.26 (0.95-1.66) | **117 (17.2)** | **95 (14.0)** | **1.37 (1.00-1.90)** |

OR: odds ratio; CI: confidence interval.

**^a^**Derived from logistic regression, conditioned on age- and sex-matched pairs, sibling pairs, or spouse pairs, respectively, after adjustment for educational attainment and household disposable income in all analyses, and additionally age and sex in the sibling and spouse analyses.

Supplementary Table 6. Previous hospital-treated infections and presentation of amyotrophic lateral sclerosis (ALS) at the time of diagnosis – univariate analysis

| Clinical characteristics | **Yes** | **No** | **P for difference** |
| --- | --- | --- | --- |
| Bulbar onset, N/total (%) | 37.0 (7.3) | 38.2 (7.1) | **0.04** |
| Family history, N/total (%) | 17.4 (21.7) | 17.6 (21.4) | 0.93 |
| ALSFRS-R at diagnosis, mean (SD) | 1.1 (1.3) | 1.0 (1.2) | 0.23 |
| Diagnostic delay in months, mean (SD) | 23.7 (4.1) | 23.6 (4.3) | 0.81 |
| Progression rate at diagnosis, mean (SD) | 129/337 (38.3) | 108/370 (29.2) | **0.01** |
| Body mass index at diagnosis, mean (SD) | 86/546 (15.8) | 92/613 (15.0) | 0.79 |
| Anxiety, N/total (%) | 36/141 (25.5) | 18/135 (13.3) | **0.02** |
| Depression, N/total (%) | 26/141 (18.4) | 11/135 (8.1) | **0.02** |
| MOCA <26, N/total (%) | 36/113 (31.9) | 36/120 (30.0) | 0.87 |
| SK-ECAS ALS specific <82, N/total (%) | 46/99 (46.5) | 44/91 (48.4) | 0.91 |
| SK-ECAS total <108, N/total (%) | 41/99 (41.4) | 43/91 (47.3) | 0.51 |
